# *Babesia venatorum* Infection in Child, China

**DOI:** 10.3201/eid2005.121034

**Published:** 2014-05

**Authors:** Yi Sun, Shao-Gang Li, Jia-Fu Jiang, Xin Wang, Yuan Zhang, Hong Wang, Wu-Chun Cao

**Affiliations:** Beijing Institute of Microbiology and Epidemiology, Beijing, China (Y. Sun, J.-F. Jiang, X. Wang, Y. Zhang, H. Wang, W.-C. Cao);; Capital Medical University, Beijing (S.-G. Li)

**Keywords:** Babesia venatorum, parasites, protozoa, human infection, child, ticks, zoonoses, China

**To the Editor:** Babesiosis, which is caused by intraerythrocytic sporozoites of the genus *Babesia*, is a tick-borne emerging zoonosis in humans. Although >100 *Babesia* species infect animals, only a few species, mainly *B*. *microti* and *B*. *divergens*, infect humans. Human infections with *B*. *microti* have been reported from the United States and other countries, and most human infections with *B*. *divergens* have been reported from Europe (*1*). Another species, *B*. *venatorum*, was found to infect humans in some countries in Europe (*2*,*3*).

Only 12 babesiosis case-patients have been reported in China, 10 of whom were infected with *B*. *microti* (*4*–*6*) and 2 with *B*. *divergens* (*7*). We report a case of babesiosis caused by *B*. *venatorum* in a child and characterize the isolated pathogen.

On April 16, 2012, an 8-year-old boy who lived in Pishan County, Xinjiang Autonomous Region, China, was admitted to Friendship Hospital in Beijing because of an irregular fever (38.6°C–41.0°C) for 12 days, anemia, malaise, myalgia, fatigue, progressive weakness, and shortness of breath. Before admission, he was given oral cefixime (80 mg/day for 5 days) at a local clinic, but no clinical improvement was observed.

At admission, the patient had a body temperature of 38.7°C, a pulse rate of 76 beats/min, a blood pressure of 110/70 mm Hg, and a respiration rate of 18 breaths/min. Laboratory tests identified hemoytic anemia (erythrocyte count 2.7 × 10^9^ cells/L, hemoglobin level 8.6 g/dL), thrombocytopenia (147 × 10^9^ platelets/L), increased levels of serum lactate dehydrogenase (1,462 U/L) and bilirubin (2.6 mg/dL), and an increased leukocyte count (17 × 10^9^ cells/L with 72% neutrophils, 24% lymphocytes, 1% monocytes, 2% eosinophils, and 1% basophils). Levels of C-reactive protein (14.2 mg/dL) and procalcitonin (3.1 mg/dL) were increased, which suggested an inflammatory process.

Forty-eight hours after the patient’s admission, a thin peripheral blood smear stained with Giemsa was prepared. A presumptive diagnosis of babesiosis was made on the basis of microscopic observation of intraerythrocytic parasites (parasitemia level ≈5%) with typical ring-like trophozoites, paired pyriforms, and tetrads (Figure, panel A).

DNA was extracted from a patient blood sample. PCR specific for a partial 18S rRNA gene sequence was performed with primers PIRO-A and PIRO-B (*8*) and showed a positive result for a *Babesia* sp. The patient was then treated with azithromycin (12 mg/kg once a day for 7 days) and atovaquone (20 mg/kg twice a day for 7 days). His clinical manifestations improved 3 days after treatment, although parasites were still detectable in blood smears. On May 17, negative results for blood smears and PCR indicated that the parasite had been cleared. The boy was discharged on May 20, 2012, and has remained healthy.

A 0.5-mL blood sample obtained from the patient before treatment was injected intraperitoneally into 3 severely combined immunodeficient mice. Mice were monitored for parasitemia every 3 days. When tested 6–9 days postinjection, all 3 mice were positive for a *Babesia* sp. (Figure, panel B). We tested for IgG against *B*. *venatorum* and *B*. *microti* by using an indirect immunofluorescence assay (*9*). Seroconversion against *B*. *venatorum* was evident; reciprocal antibody titers of 16 in an acute-phase sample (admission) and 128 in a convalescent-phase (discharge) sample. Results for *B*. *microti* were negative.

**Figure:**
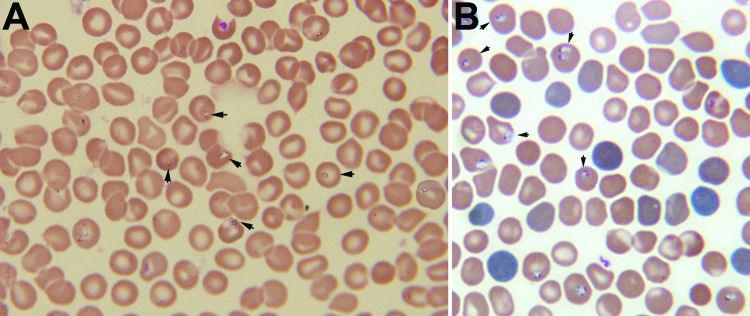
A) Giemsa-stained thin blood smear for an 8-year-old boy from China showing erythrocytes with typical ring forms, paired pyriforms, and tetrads of a *Babesia* sp. (arrows). B) Giemsa-stained thin blood smear for a mouse with severely combined immunodeficiency, which had been injected with blood from the patient, showing *Babesia* sp.–infected erythrocytes (arrows). Original magnifications ×1,000.

Nucleotide sequences of PCR products from patient and mice blood samples were identical to the corresponding sequence of *B*. *venatorum* 18S rRNA. The complete 18S rRNA gene of the *Babesia* parasite isolated from the patient was amplified with primers CRYPTOF and CRYPTOR (*2*). This PCR product was sequenced, and the sequence was submitted to GenBank under accession no. KF724377.

*B*. *venatorum* was first known as *Babesia* sp. EU1 and was named after the Latin word for hunter because the first reported infected patients were 2 occupational hunters from Austria and Italy (*2*). One human infection with *B*. *venatorum* was also reported from Germany (*3*). All 3 case-patients were men >50 years of age who had undergone splenectomies for severe Hodgkin disease before *Babesia* sp. infection.

Previously reported babesiosis cases in children have been mostly acquired by blood transfusion (*10*). The patient had no history of transfusions with blood products and had never traveled outside his home town before disease onset. Although he and his parents did not recall any tick bites, he was at high risk for exposure to ticks because he often played with his dog, which frequently went outdoors in a tick-infested forested area. The dog may have transmitted a *Babesia* sp.–infected tick to the patient. However, ticks from the dog were not available for identification and testing.

The patient in our study was presumed to be healthy and immunocompetent, which indicates that *Babesia* species can cause infections even in healthy persons. Babesiosis should be considered in the differential diagnosis of patients with a history of tick exposure and prolonged and irregular fever. Blood smear evaluation for intraerythrocytic parasites should be considered.

The patient was treated with azithromycin and atovaquone and the parasites were cleared within 1 month. This combined treatment was well tolerated and effective, and it can be recommended as an alternative treatment to the commonly used therapy of quinine and clindamycin (*1*).
